# Perception of Game-Based Rehabilitation in Upper Limb Prosthetic Training: Survey of Users and Researchers

**DOI:** 10.2196/23710

**Published:** 2021-02-01

**Authors:** Christian Alexander Garske, Matthew Dyson, Sigrid Dupan, Kianoush Nazarpour

**Affiliations:** 1 Intelligent Sensing Laboratory School of Engineering Newcastle University Newcastle upon Tyne United Kingdom; 2 Edinburgh Neuroprosthetics Laboratory School of Informatics University of Edinburgh Edinburgh United Kingdom

**Keywords:** upper limb, rehabilitation, arm prosthesis, serious games, engagement, transfer

## Abstract

**Background:**

Serious games have been investigated for their use in multiple forms of rehabilitation for decades. The rising trend to use games for physical fitness in more recent years has also provided more options and garnered more interest for their use in physical rehabilitation and motor learning. In this study, we report the results of an opinion survey of serious games in upper limb prosthetic training.

**Objective:**

This study investigates and contrasts the expectations and preferences for game-based prosthetic rehabilitation of people with limb difference and researchers.

**Methods:**

Both participant groups answered open and closed questions as well as a questionnaire to assess their user types. The distribution of the user types was compared with a Pearson chi-square test against a sample population. The data were analyzed using the thematic framework method; answers fell within the themes of usability, training, and game design. Researchers shared their views on current challenges and what could be done to tackle these.

**Results:**

A total of 14 people with limb difference and 12 researchers participated in this survey. The open questions resulted in an overview of the different views on prosthetic training games between the groups. The user types of people with limb difference and researchers were both significantly different from the sample population, with *χ*^2^_5_=12.3 and *χ*^2^_5_=26.5, respectively.

**Conclusions:**

We found that the respondents not only showed a general willingness and tentative optimism toward the topic but also acknowledged hurdles limiting the adoption of these games by both clinics and users. The results indicate a noteworthy difference between researchers and people with limb difference in their game preferences, which could lead to design choices that do not represent the target audience. Furthermore, focus on long-term in-home experiments is expected to shed more light on the validity of games in upper limb prosthetic rehabilitation.

## Introduction

### Background

Serious games have been shown to enhance the outcome of movement rehabilitation after stroke or cerebral palsy [1,2]. Computer games have also been researched for upper limb prosthetic control training since the early 1990s [3-14]. Although the field has received considerable attention over recent years, it has yet to succeed in finding general proof for successful transfer of training myoelectric control in the games to an increase in prosthetic control ability [15]. For instance, transfer has only been found in training a strongly activities of daily living (ADL)–relevant task in a virtual environment [15].

The skills required to play a game can be learned with practice. A serious game incorporating training exercises consistent with a rehabilitation regimen allows the numerous repetitions of exercises necessary during rehabilitation therapy to take place. Nonetheless, it is hypothesized that the training needs to be engaging to ensure regular and consistent adherence to the exercises [12,16]. Current prosthetic training games revolve around 2 core themes: the engagement of the player [7,8,12,14,16-20] and the skill transfer from the game to prosthetic use [15,20,21]. These 2 objectives are pursued with 2 types of video games that aim to improve myoelectric control (*myo-games)* [10] in prosthetic training: (1) games that are based on existing game platforms with input adjusted to the electromyogram (EMG) signals [4,6] and (2) completely novel games around specific training goals [9,20].

Few researchers specifically acknowledge the importance of considering the diversity of the target audience when developing games in general [16,17,22,23] and, therefore, myo-games specifically. Even fewer researchers have attempted to address this difference in preferences in their developed games and take the views of people with limb difference into account before starting the development process. A notable exception is the work of Tabor et al [16], who collected qualitative feedback from a small testing group and incorporated the suggestions into their game design. Owing to the diversity in the population, where people have varying definitions of fun aspects and expectations for games, multiple attempts have been made to categorize people into user types [24-26]. Professional game development is based on the psychology behind those types of gamers and the choice of the appropriate game design elements fitting for the target audience. However, academic game development is typically carried out over a relatively short period and by a small, nonspecialist team, which is in stark contrast to the years of development time often carried out by a large and highly specialized team that goes into modern games. This means that the decision making in these games is potentially subject to the preferences of a small team that does not generally reflect the preferences of the target audience.

### Objectives

We hypothesized that there are considerable differences between the views of prosthesis end users and researchers with regard to engaging aspects of a game. To test this hypothesis, we created a survey and sought to determine the focus points of each of these groups. In addition, we included a user type questionnaire to ascertain the distribution of each participant group for comparison. Using such a user type distribution can deliver useful information to lead general design choices in game development. It could also be used for presets that emphasize certain game design elements over others for increased user engagement. Furthermore, this survey aimed to identify other challenges than potential disparities in game preferences that the community of researchers could have to face on the path of game-based upper limb prosthetic rehabilitation. This study adds the opinions of researchers and people with limb difference about games in upper limb rehabilitation to the research that has been conducted on the opinions of clinicians [27].

## Methods

### Study Design

The study was approved by the University Ethics Committee of Newcastle University under the reference number 905/2020. The survey was conducted from February 2020 to May 2020. Participants were either people with upper limb difference or researchers who were active in the research of games for prosthetic training or in prosthetic research in general. All participants gave their consent by filling out and submitting the survey as stipulated on the first page of the survey form.

The recruitment of this study was conducted predominantly on the web via personal contacts. Additional outreach was done via social media and by contacting charities in the United Kingdom that are involved with people with limb difference. Specifically, the survey was sent out to the main and local branches of 13 different charities as well as 40 researchers involved in upper limb prosthetic research. The inclusion criterion for people with limb difference was the absence of the upper limb, irrespective of level, side (unilateral or bilateral), or use of a prosthesis. A total of 14 people with limb difference and 12 researchers filled out the survey. An overview of the demographic data of the participants is presented in Tables 1 and 2. We chose a web-based survey and expected that the web-based nature would increase the number of people willing to participate because of ease of access. In addition, the web-based survey offered the participants time to think about their answers without the pressure of coming up with an answer on the spot. The survey was developed in English and was not altered over the course of the study. The participants were given the option to contact the authors if they did not want or were not able to fill out the survey on the web. No participant used this option.

The survey first introduced the general aim of the study and the contact information of the first and the last author and the Data Protection Officer of Newcastle University. The survey asked for general demographics and, in case of people with limb difference, for anamnesis with regard to their limb. This was followed by a user type questionnaire originally developed by Tondello et al [26]. The survey concluded with open questions about the preferences and opinions of participants with regard to games in general and games in prosthetic training specifically. The researchers were asked to answer additional questions concerning the challenges in this field of research.

**Table 1:** Participants’ demographics.

Gender	Age group (years)	Type of limb difference	Side of limb difference	Level of limb difference	Prosthesis use	Former participation in research
F^a^	31-40	A^b^	Dominant	Above elbow	No, but interested	Yes
M^c^	31-40	C^d^	Dominant	Below elbow	Former	No
F	41-50	A	Both	Below elbow	Tried	No
F	41-50	A	Nondominant	At shoulder	No, but interested	No
M	41-50	C	Nondominant	Below elbow	Active	Yes
F	41-50	A	Dominant	Below elbow	No, but interested	No
M	41-50	C	Nondominant	Below elbow	Active	No
F	41-50	A	Dominant	Above elbow	Tried	Yes
M	51-60	A	Dominant	Below elbow	Active	Yes
F	51-60	A	Dominant	Below elbow	No, but interested	Yes
F	51-60	C	Nondominant	At wrist	No, no interest	Yes
M	51-60	C	Nondominant	Below elbow	Active	Yes
M	51-60	A	Dominant	Below elbow	No, but interested	No
M	61-70	A	Nondominant	Below elbow	Active	No

^a^F: female.

^b^A: amputation.

^c^M: male.

^d^C: congenital.

**Table 2:** Researchers’ demographics.

Gender	Age group (years)	Profession
M^a^	20-30	Researcher
M	31-40	PI^b^
M	31-40	Medical doctor
M	31-40	PI
M	31-40	PI
M	41-50	PI
M	41-50	PI
M	41-50	PI
M	41-50	PI
M	41-50	PI
Undisclosed	Undisclosed	PI
Undisclosed	Undisclosed	Undisclosed

^a^M: male.

^b^PI: principal investigator.

### Data Analysis

The results of the user type questionnaire were processed using MATLAB (The MathWorks, Inc). A goodness-of-fit test using the Pearson chi-square test with a significance level of α=*.*05 was conducted for both participant groups, compared with the distribution published by Tondello et al [26]. This test was chosen to identify potential differences between the distribution of user types of the participant groups and the distribution of a larger sample population.

For the analysis of the resulting data for the open questions of the survey, we applied the thematic framework approach [28]. This approach consists of 5 steps:

1. Familiarization: All authors familiarized themselves with the collected data. The first author created an initial theme set, which was discussed and agreed upon by all authors.
2. Identifying a thematic framework: The first author created a set of subthemes for the data set. This was approved by the last author for use in the next steps. The full set of themes can be found in Table 3.
3. Indexing: All authors coded the interview data independently. These were discussed between all authors until a consensus was reached.
4. Charting: The data were sorted by themes and subthemes by the first author.
5. Mapping and interpretation: The first author summarized and interpreted the charted data according to the themes.

**Table 3:** Thematic framework.

Themes		People with limb difference (n=39), n (%)	Researchers (n=108), n (%)
**Usability**		*6 (15.4)* ^a^	*11 (10.2)*
	Accessibility	4 (10.3)	4 (3.7)
	Data management	0 (0)	2 (1.8)
	Hardware	2 (5.1)	5 (4.6)
**Training**		*14 (35.9)*	*38 (35.2)*
	Muscle development and control	4 (10.3)	4 (3.7)
	Prosthetic ability	7 (17.9)	15 (13.9)
	Additional benefits for users	2 (5.1)	8 (7.4)
	Clinical and research benefits	1 (2.6)	4 (3.7)
	Education	0 (0)	4 (3.7)
	Feedback	0 (0)	3 (2.8)
**Game**		*19 (48.7)*	*32 (29.6)*
	Affect	5 (12.8)	17 (15.7)
	Personalization	3 (7.7)	7 (6.5)
	Social aspects	0 (0)	1 (0.9)
	Mechanics	11 (28.2)	7 (6.5)
**Challenges**		—^b^	*27 (25.0)*
	Justification and reasoning	—	11 (10.2)
	Design and development	—	10 (9.3)
	Involvement	—	2 (1.8)
	Recognition	—	4 (3.7)

^a^Italicized values denote the subtotals for the respective main themes.

^b^—: Not available. People with limb difference were not asked about research-specific challenges.

## Results

### General

A total of 14 people with limb difference and 12 researchers in the prosthetic field participated in the survey (Tables 1 and 2, respectively). Of the 14 people with limb differences, 7 had experience with studies pertaining to rehabilitation with a computer-based device before the survey.

An overview of the themes identified from the survey data is presented in Table 3. In the following sections, the responses of the survey participants are presented. The texts in *italic* font are direct quotes from the participants. The results are reported thematically: usability, training, game design, and challenges. The raw survey results are provided in Multimedia Appendix 1.

### Usability

**Figure 1:**
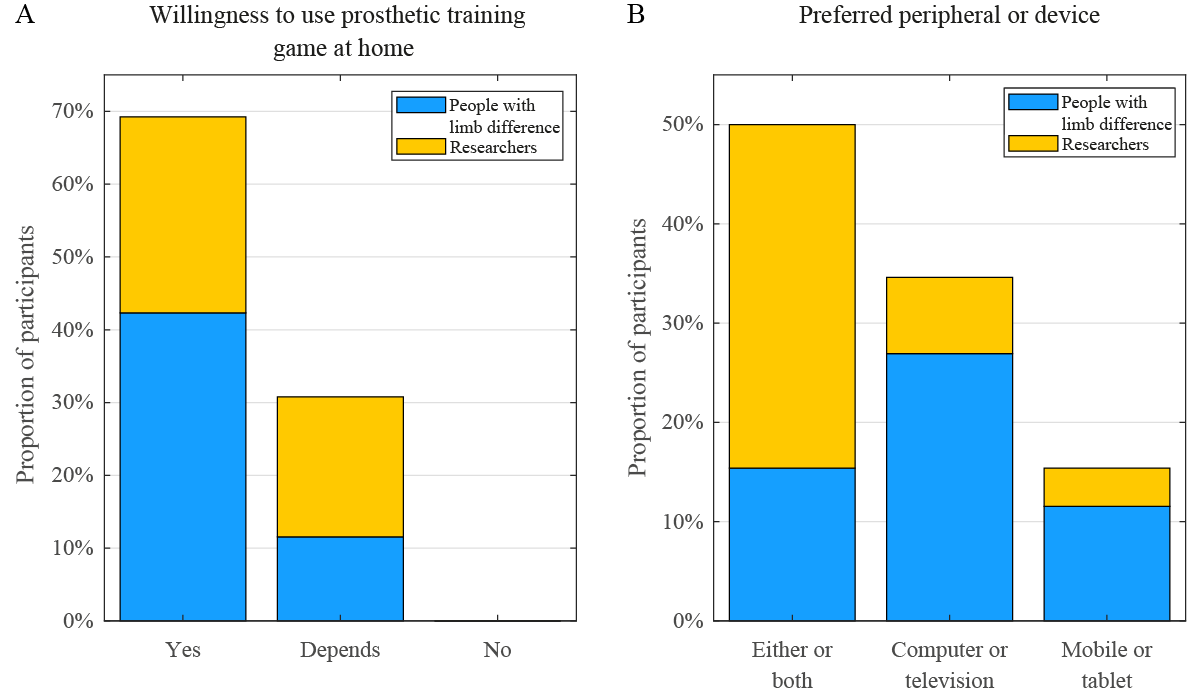
Participant response proportions for (A) their willingness to use a game-based prosthetic training tool at their own home and (B) their preferred peripheral or device for such a game.

The majority of the participants, both people with limb difference and researchers, indicated that they believed that people in prosthetic training would use game-based training at home (Figure 1). A total of 3 of the 14 people with limb difference and 5 of the 12 researchers cautioned that factors predominately concerning usability and game design can affect adoption, as usability is one of the issues as to why former gamers have stopped playing virtual games:

I used to play an Atari, as it had joystick controls, but I struggle with handsets, so [I have] not played for some time.

In addition, participants pointed out that such a tool should prove easy and robust to use both in setup as well as in the actual use of the tool.

An additional concern was raised with regard to the compatibility of the software with potentially existing hardware at home. One participant said:

[Requirements for game-based prosthetic training include] providing a setup that is easy to use and robust; ensuring a game framework that allows people to use their own devices.

Creating a game framework that would work cross-platform with potentially outdated hardware is a significant challenge for the myoelectric control research community. A step in this direction could be the development of web-based gaming platforms, as mentioned by one of the researchers. Furthermore, researchers have indicated the importance of the exchange of code and knowledge within the community.

The preferred choice of platforms and peripherals, the input and output devices with which the game is played, was more distinct in the case of the participants with limb difference than the researchers. Figure 1 shows that the majority of people with limb difference would prefer a computer or television screen. However, researchers have shown no clear preference for the mobile over fixed screen options.

### Training

The topics connected to the main theme of training were grouped into muscle development and control; prosthetic ability; additional benefits for users, clinicians, and research; education; and feedback.

The answers concerning muscle development and control mostly centered on the participants’ hopes and expectations. People with limb difference expect the game to help develop the musculature in their remaining limb and improve prosthetic dexterity with these muscles. For instance, a participant with limb difference wished for a game that highlighted the following:

my ability to use advanced prosthetics.

Another participant with limb difference would like to see a game made that also helps to reduce the phenomenon of phantom limb pain.

Researchers additionally specified their hopes in the direction of accelerated learning speeds, the training of ADL-related tasks, and the transfer of the skills learned in the game to prosthesis use. One researcher stated that any tool created should be based on the needs of the patients as well as clinicians. Outside of the physical benefits, researchers pointed out that a game-based tool could allow the user to share data with other users, if they wanted to, and potentially lead to a reduced need for input through in-clinic appointments.

In addition, a training game could support therapists in assessing patients’ abilities. One researcher indicated:

In the end the games should also relieve the therapists from time spending on rehabilitation. Moreover, the games should be helpful as an assessment tool for the appropriateness of a prosthesis for a given patient.

The need of personalized feedback for users was raised by a couple of researchers. They envisioned the benefits of regular and individualized training progress feedback by which users as well as therapists can track their skill progression properly.

### Game Design

The answers of the participants directly relating to design choices for the game were grouped under the main theme of game design. The subtheme of affect included all answers that aimed at an emotional response of the user; personalization contains in-game control of design for the player; social aspects revolve around the possibility of interaction with other players; and answers in the mechanics subtheme pertain to ideas for the mechanical features of the game.

With regard to the affective aspect of the design, participants of both groups indicated that the game had to be engaging for in-home adaption. For instance, one researcher points out that rehabilitation games should not only be interesting in the short term but also be able to secure the long-term engagement of the user. However, engaging the user to a sufficient level was recognized as a challenge.

Furthermore, researchers say that the game should be attractive to users, although it could be hard to appeal to a wide range of potential users. A range of games or in-game options could be beneficial because of the differing motivating factors. The degree of immersion was wished for by a participant with limb difference, and another participant pointed out the potential of an *empowering portrayal of life postamputation* in the game, for example:

That the game would be so immersive that the participant would not realise they are training their remnant muscles for optimal EMG based control.

Interestingly, one participant with limb difference suggested an *end of world* setting. As this might not feel appealing for all users, the setting should be customizable by the player. Some participants from the limb difference group wanted to see a relatable character using a prosthesis in the game, tying in with the empowering effect mentioned earlier. This affords a viable option for personalization:

Perhaps the protagonist could be a prosthetic wearer

...

The variety of preferences in the thematic setting of the game can be seen in Multimedia Appendix 2. These are results of a multiple-choice question and therefore do not add up to 100%.

The mechanics of a game-based prosthetic training tool are predominately addressed with respect to the game genre. Participants of the limb difference group identified a variety of different genres as desirable, including quiz and puzzle games, but also adventure, shooter and fighting games, and horror games. One participant mentioned more specific activities, such as camping, fishing, and shooting. A researcher argued against the use of war and fighting mechanics in clinical settings. The different genre preferences of the participants can be found in Multimedia Appendix 2. As mentioned earlier, these results do not add up to 100%.

An additional influence on the type of game and the game mechanics involved can be the type of gameplay the user prefers. As it cannot be assumed that a user is an active or former gamer and therefore knows what they look for in a game, an assessment of the user types using the Hexad Scale [26] was conducted in this survey. The outcome of this assessment for both people with limb difference and researchers can be seen in Multimedia Appendix 2. Among the people with limb difference, the philanthropist and the achiever user type are tied as clearly the most common types, whereas the player and the disruptor are the least commonly occurring. None of the researchers were grouped into players and disruptors, which is similar to them being the least common type in the other group. We observed a notable difference in the overall distribution of user types to people with limb difference. A Pearson chi-square test was conducted to test the statistical significance of this difference. The results showed that both groups were significantly different from the control group in a study by Tondello et al [26] at a significance level of α=*.*05. However, the results for the people with limb difference at *χ*^2^*_5_*=12*.*3 are noticeably closer to the critical value of *χ*^2^_5_=11*.*1 than the result of the researcher participant group of *χ*^2^*_5_*=26*.*5. This indicates that the group of participants with limb difference showed a higher similarity to the sample population presented in a study by Tondello et al [26] than the group of researchers showed to the same sample population.

Apart from the genre, people with limb difference indicated that they would like a progressive and appropriate increase in difficulty, would like to use both hands to play the game, and would like for the game to motivate them to make enough repetitions of the trained arm to form habits. A researcher pointed out that the abstraction of the signals to rewarding or menacing game elements could be beneficial.

### Challenges

Only researchers participated in this part of the survey. They were asked to formulate their opinions on the challenges that the field of games in upper limb prosthetic rehabilitation faces. In addition, they were invited to propose potential actions that could be undertaken by the community to address these challenges.

Part of the challenges mentioned by the researchers was the justification for the use or the development of serious games in prosthetic rehabilitation. The meaningful impact in terms of skill transfer to prosthetic use by myo-games must be investigated. This was stated not only for short-term effects but also for long-term benefits, when compared with other rehabilitation methods. The recognition of the difference between in-game improvement and actual benefit for prosthetic use has not yet been widely acknowledged:

However, most game studies focus only on in-game improvement. Now in-game improvement is a requirement for transfer to daily life performance. However, in-game improvement is not a sufficient requirement for transfer.

Therefore, researchers have called for longitudinal and large-cohort studies in the field to show the appropriateness of the medium used and the transfer capabilities of—potentially only certain types of—games.

The development of these myo-games faces its own problems and challenges. They should make the benefits clearly visible for the user but at the same time make the training imperceptible by shifting the focus of the user away from the underlying reason for the training onto the task-specific in-game goals. Researchers have indicated that the formation of bad habits to win the game by potentially compromising the training efficiency should be avoided. Therefore, it was recommended to involve game developers in the process and to parallelize game development and transfer testing procedures to avoid losing sight of either aspect. One researcher proposed the development of a knowledge and information sharing platform for myo-games. He also indicated that such a platform could lead to a wider and easier access to developed games for users at home with their existing hardware.

Finally, the recognition of the value of myo-games is another key obstacle to tackle. Some people, especially certain age groups, might dismiss games as frivolous and a waste of time. Both clinicians and patients might need to be convinced that a serious game for prosthetic rehabilitation could benefit them as well as potentially benefit research. For example, a researcher suggested:

Educating participants about serious games and why the time they spent playing is well spent

...

**Figure 2:**
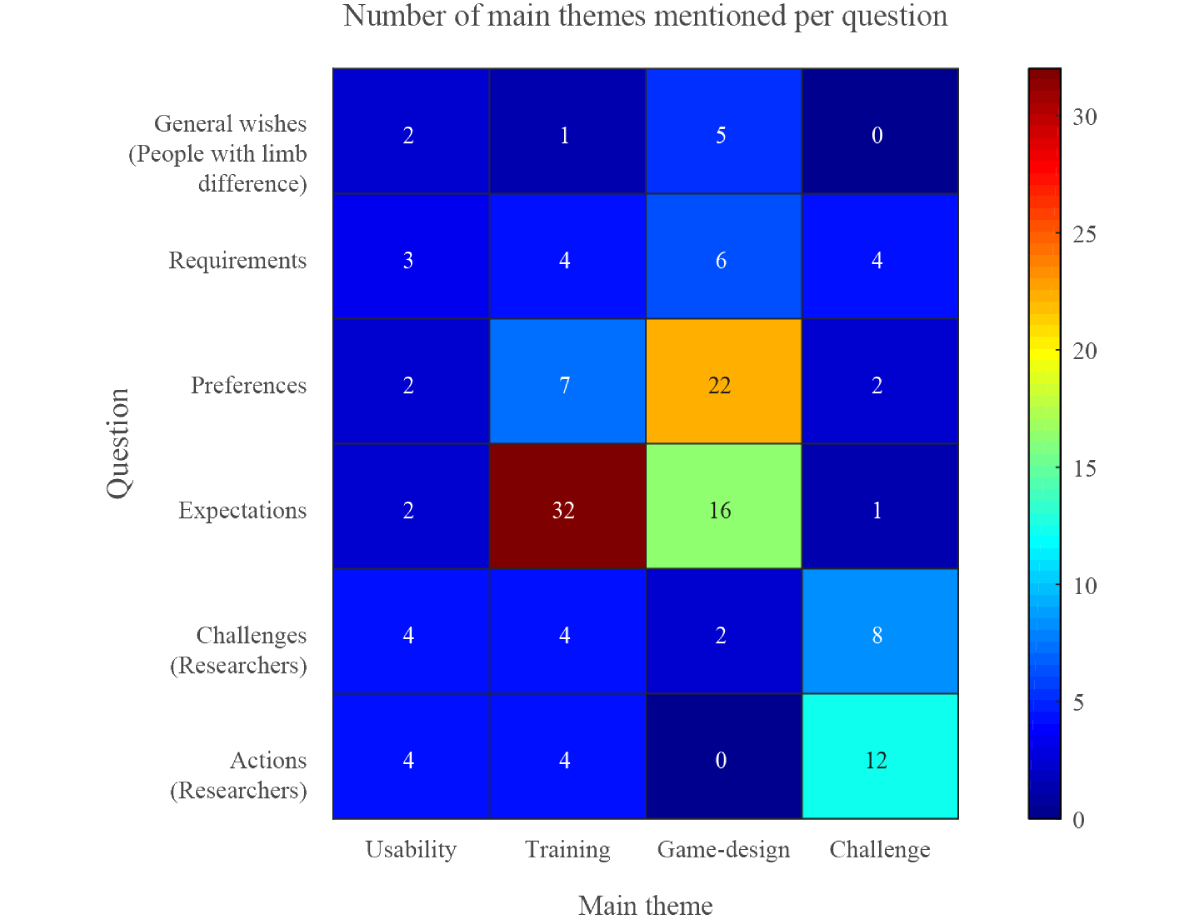
Number of coded responses per question and main theme over both participant groups.

Reviewing the number of mentions of the main themes, a clear separation of themes becomes visible. In Figure 2, it can be seen that the main expectation of the participants for the prosthetic training lies within the training benefits that it should provide. The fewer mentions of game design topics indicate that the design is of concern but that the training aspect takes priority and should be the base minimum of any game-based training. The themes of training and game design almost have the same amount of mentions overall in this survey, with 52 and 51 responses, respectively. In contrast to the clear focus on training in the expectations, the game design–related themes were more split between preferences expectations. This suggests that many game design traits are considered desirable but not a necessary component, which is reflected in some research in the field of game-based prosthetic training.

## Discussion

### Myo-Games: Opportunities and Challenges

The aim of this study is to determine the preferences, expectations, and views of both researchers and people with limb difference on game-based prosthetic training. The outcome of this survey indicates a general willingness and tentative optimism toward the topic. However, for the wide adoption of myo-games, several scientific and engineering challenges should be addressed.

In the following sections, we will discuss all identified challenges in the context of usability, training, and game design themes.

### Usability

With respect to hardware usability, the discontinuation of the commonly used Thalmic Myo Gesture Control armband [9,13,20] means there is now a lack of low-cost, easy-to-use EMG sensors for home use. Of the commercially available dry EMG options suitable for game-based training, almost all require sensors to be accurately positioned using adhesives. As such, researchers are increasingly developing custom-built EMG acquisition solutions for game-based systems [14,18,29]. This approach does not scale well and is likely to contribute to the slow translation of laboratory research to translational research with a larger number of participants.

The accessibility of in-home myo-game software is also a key factor for overall usability. Accessibility must be balanced against client privacy and patient confidentiality, key points that therapists have identified as important in game-based upper limb rehabilitation [27]. The small target population of upper limb amputees and the niche nature of game-based rehabilitation mean cross-platform software with widespread hardware compatibility is not likely to be financially viable. The low cost of Android mobile devices and the fact that they can be locked down to a restricted set of sandboxed apps make this platform the most likely candidate for in-home use.

### Training

The topic of training with regard to goals was fairly unanimous between the participants with limb difference and the researchers. In current research in this field, it appears to have mostly been assumed that an improvement in game control would translate readily to an improvement in prosthetic control as only in-game improvement or abstract control has been measured in most studies [4,6,8,12-14,20,30,31]. The question of whether this transfer can happen and with which type of game this might happen has yet to be answered, as current research challenges the idea that a general myocontrol skill exists [10,32]. However, it could prove beneficial to not only consider direct transfer but to also investigate indirect improvements like increases in training speed in learning the use of a prosthesis after training with myogames. Therapists have especially expressed that gaming in therapy should be balanced with other forms of therapy [27], and as such, it might prove beneficial to examine them in conjunction.

The only testing of the effect on actual prosthetic skill in game-based prosthetic training research was conducted on the direct effect that the developed game would have on prosthetic skill [10,15]. We could not find any study that has conducted research if the training with a game before or alongside actual training with a prosthesis could be beneficial to the learning process as opposed to an immediate effect on prosthetic skill. Prior training could be sensible as post amputation, the site of surgery may not yet be ready for the fitting of a prosthetic socket. The British Society for Rehabilitation Medicine states that the fitting may be deferred from 4 to 6 weeks after the amputation [33]. In both the amputees and people with congenital limb difference, the muscle sites could need development before a prosthesis can be considered. Training alongside real-life prosthetic training could add to the fine control without being reliant on the other arm musculature when those muscles are tired from the weight of the prosthesis.

In contrast to the popular assumption that a prosthetic training game would reduce the time investment necessary for the therapist, it should be pointed out that this is highly dependent on the type of training tool and the way it is included in the exercise regime. Almeida and Nunes [34] point out that therapists might need to be involved in the setup and fulfill a supervisory role in the exercises performed by users. Optimally, this would not be the case after the system was tested and approved apart from a first introductory session with the therapist to ensure that the user understands how to use the system properly. Therefore, ease of use is an important factor, both with respect to setting up equipment and its daily use.

Responses from the participants with limb difference group indicate that they would like a prosthetic training tool that makes rehabilitation feel less like rehabilitation. This was reflected in the call of the researchers for immersion in the game, which allowed the user to shift from their limb and muscles to the task at hand, allowing the motions to become intuitive. This can also be found as part of the Optimizing Performance Through Intrinsic Motivation and Attention for Learning theory of Wulf and Lewthwaite [35], which states that an external focus has beneficial effects on motor learning as well as the sense of accomplishment. According to that theory, the external focus as well as intrinsic motivation feed into a virtuous cycle of enhanced motor learning. However, the question of how to incorporate these aspects efficiently is yet to be answered.

### Game-Design

One of the main points mentioned for the game design was the facilitation of both short- and long-term engagement. The potential users of a prosthetic training tool seem willing to take the leap to use it, which is supported by research in games for other conditions [36], but the appeal of such a novelty can quickly wane. It is the task of good game design to support the user by providing motivating gameplay for the entire training period. This again feeds into the aforementioned virtuous cycle, according to Wulf and Lewthwaite [35]. A shared experience among prosthesis users as well as between prosthesis users and able-bodied people could further enhance the motivation and potentially the training intensity [36-38].

The involvement of all stakeholders, including game developers, is a recommended path for research to take. This can be especially motivated, as game developers have more experience in catering toward a specific audience with their games and which game design elements work best to keep the users motivated to play the game. Academic teams are likely to have different views on fun themes and activities to the general population based on the differences in the user types in this study. These differences further support the need for co-design between researchers and potential end users [17,29,39]. The inclusion of the design preferences of the users as well as the input by their families can provide valuable insights for the researchers developing the games [40]. In addition, an understanding of the practical activities of the therapists and their involvement in game-based rehabilitation is necessary for the effective development of a training tool that benefits all parties [34]. As of yet, cocreation in the wider field of prosthetics remains difficult to integrate with current academic methods but is now becoming a focal point for translational research [Jones et al, forthcoming].

Nonetheless, this gives rise to the question of how to achieve effective targeting of the game in a highly varied target audience. Working toward increasing the engagement of a game before knowing whether transfer will happen for this game could lead to fruitless efforts. However, it is possible that engagement is a contributing factor in the transfer process and therefore worthy of further exploration.

As the current state of myo-game development is very diverse, it was expected that the opinions on the matters to focus on and the potential resolutions are just as varied.

### The Survey: Strengths and Limitations

It is the first time a survey like this has been conducted in this field with these participant groups. We did not include clinicians in this survey, as a previous study covered therapists’ views on the use of video games in general upper limb rehabilitation [27]. The format of the study allowed the easy spread of the survey in the field. This led to the recruitment of 12 researchers and 14 participants with limb difference. However, the sample size of the survey was smaller than would be desirable for a good cross-section of the population with limb difference as well as researchers. In an attempt to acquire many participants, this survey has been spread in various ways, but it is possible that survey fatigue has stopped people from participating. This could indicate that other means of interacting with people with limb difference might be advisable for future research. If a similar study was to be repeated in this format, we recommend collaborating on it with several research groups. This would provide access to a larger group of researchers as well as people with limb difference.

In addition, regarding the data set of the researchers, the clear majority of participants identifying as male could influence the outcome of the answers. Female researchers were included in the list, and the survey was distributed among them. However, because of the general gender disequilibrium in the field, this list already contained a higher percentage of male researchers. This could have influenced the opinions and preference distribution of the researchers, as differences in gaming preferences have been identified in both gamer and nongamer populations [41].

### The Future of Myo-Games

In this study, the results indicate a higher level of similarity of the participants with limb difference group to the sample population in a study by Tondello et al [26] than of the researcher participant group compared with the same sample population. Although the low number of participants does not provide conclusive evidence in this matter, it is still worth considering the implications of this dissimilarity. The influence of personal preferences and assumptions made by the researchers could have a significant impact on the engaging and motivating aspects of the game they are developing. This could be mitigated by bringing in professional support from the game development sector or by increasing collaboration with game experts within academia, who have more practical knowledge in catering a game experience toward a diverse target audience. Moreover, this would likely benefit the efficient incorporation of features facilitating external focus and intrinsic motivation. However, as the market for serious games for upper limb prosthetic rehabilitation is fairly small and therefore the potential profit margin is small, including professional game developers in this research could prove challenging. In addition, involvement of the users, their families, and other stakeholders can provide additional benefits to the development process and should be considered.

Furthermore, the effect of myo-games has mostly been investigated in short trials of up to a week in experimental scenarios at a university or at the home of the participant. A much-improved assessment of the effect of these games in both the short term and the long term as well as whether a significant level of transfer occurs could be achieved by conducting longer-term home trials with people with limb difference. This would also provide more information about the engaging aspects of the game in the long term, that is, if the feelings of the participants change because of the waning novelty of the game and if it becomes a chore. Conversely, if the attrition over a long-term study would prove to be significantly lower than comparable experiments, this could be an indicator for a positive effect of games on exercise and training adherence. As discussed, for long-term experiments in a home environment, Android mobile devices appear to be a promising choice for hardware. In addition, the effect of myo-game training in conjunction with conventional prosthetic training could prove beneficial to assess.

The current state of isolation because of the 2020 coronavirus pandemic poses many challenges for academic research, some of which may persist in the long term. Thus, a shift in experimental procedures to the home environment of the participants could be a beneficial route to follow. Many of the technical challenges surrounding home-based experimentation, such as precision timing, low latency networking, and data security, have already been widely addressed in gaming. More widespread adoption of gaming technology to facilitate the shift of experimentation to participants’ homes may provide an alternative route to bridge the gap between academic research and viable prosthesis training solutions.

## Appendix

Multimedia Appendix 1 Questions and results.

Multimedia Appendix 2 Preferences and user type distributions.

## Abbreviations

ADL: activities of daily living
EMG: electromyography
