# Supplementary material for: Perception of Game-Based Rehabilitation in Upper Limb Prosthetic Training: Survey of Users and Researchers
Source: JMIR Serious Games. 2021 Feb 1;9(1):e23710. doi: 10.2196/23710 (PMC7884217; doi:10.2196/23710)
Supplement: Multimedia Appendix 1 [file games_v9i1e23710_app1.pdf]

1 Survey

1.1 Page 1: Personal Questions

Table 1: List of the personal questions for limb different people (LD) and principal investigators (PI) and the available answer options. The asterisk marks a required field.

Field	Options	LD	PI
Name	[Open]	✓	✓*
Year of Birth	[Open]	✓*	✓*
Gender	Female, Male; Prefer not to say; Prefer to self describe as [open]	✓*	✗
What type of limb difference do you have?	Acquired; Congenital	✓*	✗
How much time has passed since your limb loss?	0-6 months; 7-12 months; 13 months to 3 years; More than 3 years	✓*	✗
On which side is your limb difference located?	Dominant arm only; Non-dominant arm only; Both arms	✓*	✗
What is the degree of your limb difference?	Hand/Partial Hand; At Wrist; Below Elbow; At Elbow; Above Elbow; At Shoulder; None of the Above	✓*	✗
Do you have experience with a muscle-controlled prosthesis (a myo)?	None, but I would like to; None, and I have no interest in using one; Yes, I have tried one before; Yes, I was an active user but not anymore;Yes, I use one actively	✓*	✗
Have you taken part in a study of computer-based rehabilitation before (in form of a game, a simulation, a virtual reality experience, etc)?	Yes; No	✓*	✗
What is your profession?	[Open]	✗	✓*

1.2 Page 2: User Type Questionnaire

Table 2: Hexad scale statements and their associated user type.

Statement	User Type
I like to provoke.	Disruptor
I like being part of a team.	Socialiser
It is important to me to always carry out my tasks completely.	Achiever
It is important to me to follow my own path.	Free Spirit
I like competitions where a prize can be won.	Player
The wellbeing of others is important to me.	Philantropist
Rewards are a great way to motivate me.	Player
I like to question the status quo.	Disruptor
I often let my curiosity guide me.	Free Spirit
It makes me happy if I am able to help others.	Philantropist
I like defeating obstacles.	Achiever
It is important to me to feel like I am part of a community.	Socialiser
If the reward is sufficient I will put in the effort.	Player
I enjoy group activities.	Socialiser
Being independent is important to me.	Free Spirit
I like helping others to orient themselves in new situations.	Philantropist
I like mastering difficult tasks.	Achiever
I dislike following rules.	Disruptor
Interacting with others is important to me.	Socialiser
Return of investment is important to me.	Player
I like to try new things.	Free Spirit
It is difficult for me to let go of a problem before I have found a solution.	Achiever
I see myself as a rebel.	Disruptor
I like sharing my knowledge.	Philantropist

1.3 Page 3: Preferences and Opinions

Table 3: List of the preferences and opinions asked of limb different people (LD) and principal investigators (PI) and the available answer options (if provided). The asterisk marks a required field.

Field	Options	LD	PI
What thematic setting do you enjoy? (in games, books, movies, TV shows, etc) (multiple answers are possible)	Crime; End of the World; Fantasy; Food; History; Holidays; Law; Medicine & Science; Nature; Politics; Science-Fiction; Sports; Supernatural; Travel & Transportation; War & Fighting; Other [Open]	✓*	✓*
Do you play video games or did you play them in the past?	Yes; No	✓*	✓*
Which genre of video games did/do you enjoy? (multiple answers are possible)	Action; Adventure; Racing; Fighting; Puzzle; Roleplaying Games; Shooter; Simulation; Sports; Strategy; Other [Open]	✓*	✓*
Would you be interested in playing video games?	Yes; No; Not Sure	✓*	✗
What video games would you like to be able to play?	[Open]	✓*	✗
What kind of game would you like developers to make? (looks, genre, setting, etc)?	[Open]	✓*	✓*
Would you use a game for prosthetic training at home if you had the option?	Yes; No; Depends	✓*	✗
Do you think a game for prosthetic training at home would be used by patients?	Yes; No; Depends	✗	✓*
What would the use of a game for prosthetic training depend on?	[Open]	✓*	✗
What do you think the use of such a game for prosthetic training would depend on?	[Open]	✗	✓*
What screen would you prefer to train on?	Computer/TV Screen; Mobile/Tablet; Either/Both	✓*	✗
What device do you deem more appropriate for a training tool?	Computer/TV Screen; Mobile/Tablet; Either/Both	✗	✓*
What hopes/expectations would you have for such a training game?	[Open]	✓*	✓*
In your opinion, what are the current challenges in terms of using games in prosthetics?	[Open]	✗	✓
What are specific actions that could be taken by us as a community to address these challenges?	[Open]	✗	✓

2 Results

2.1 Multiple-choice Questions

Table 4: Results of multiple-choice question regarding preferred game genre.

Genre	Limb Different	Researchers
Action	3	3
Adventure	3	4
Fighting	2	2
Puzzle	3	2
Racing	4	2
Roleplaying Games	1	2
Shooter	3	5
Simulation	0	1
Sports	1	2
Strategy	1	5

Table 5: Results of multiple-choice question regarding preferred game setting.

Setting	Limb Different	Researchers
Crime	11	8
End of the World	3	3
Fantasy	3	4
Food	5	5
History	5	9
Holidays	6	2
Law	1	2
Medicine& Science	5	10
Nature	6	9
Politics	2	6
Science-Fiction	4	5
Sports	6	4
Supernatural	3	1
Travel & Transportation	4	7
War & Fighting	5	3

2.2 Closed Questions

Table 6: Results of closed questions.

**Interest in playing video games**	**Limb Different**	**Researchers**
Yes	5	-
No	3	-
Maybe	6	-

**Current or Previous Gamer**	**Limb Different**	**Researchers**
Yes	7	7
No	7	5

**At-Home Game-Based Prosthetic Training**	**Limb Different**	**Researchers**
Yes	11	7
No	0	0
Depends	3	5

**Preferred Device/Peripheral**	**Limb Different**	**Researchers**
Computer/TV	7	2
Mobile/Tablet	3	1
Either/Both	4	9

2.3 Open Questions

2.3.1 General Wishes: What video games would you like to be able to play? (Limb Different)

- I enjoy quiz game.
- Any virtual game 2 handed
- don't know
- I used to play an Atari, as it had joystick controls, but I struggle with handsets, so not played for some time.
- 1st person shooter
- I used to enjoy playing BioShock, Hitman,Resident evil style games
- Puzzle
- Ones that would help me understand hand movements

2.3.2 Requirements for Home Use

Limb Different: What would the use of a game for prosthetic training depend on?

- Ease of use. Interesting or engaging. Realistic
- What the game was and entailed.
- On the game, on ease of set-up, and on compatibility with my somewhat old laptop (2010)!

PIs: What do you think the use of such a game for prosthetic training would depend on?

- To be short, the intrinsic motivation of the patient to improve control, the trust of the patient in the system that engaging in the game will lead to meaningful impact, the positive feedback available and visible progress to the patient in their improvement, and how fun the game is. Off the top of my head those are the leading factors that are highly interconnected.
- The game would need to be engaging, and the user would need to see the benefits of engaging in a reasonable time frame.
- If the user played video games or not.
- The engagement of the user and the choice of paradigm
- 1. how exciting it is
  2. how effective it is afterwards

2.3.3 Preferences: What kind of game would you like developers to make? (looks, genre, setting, etc)?

Limb Different:

- Yes.
- More adventure games, camping, fishing, shooting included
- don't know
- An end of the world/ shooting/fighting would be interesting.
- Not sure
- An immersive style game that progressively gets harder - perhaps the protagonist could be a prosthetic wearer (Mad max/ metal gear style?! http://www.nuskool.com/learn/lesson/the-future-of-prosthetics-metal-gear-inspired-arms/) - an empowering portrayal of life post amputation.
- Puzzle
- Settings

Researchers:

- Something that is appropriate to the target audience. A game for children might not be suitable for adults... depending on the design. Something that is of good quality.
- no preference about this
- no war/fighting games
- This depends on the individual patient, as different age groups and individuals will find different motivating factors. May be best to have a range. Or, maybe the question is what time of game to develop, but how can the interface be developed to play the current range of games available across mobile, desktop and console gaming platforms. Cosima Prahm in Vienna is a good person to talk to on this, as has already developed some games for this purpose, so I would hope that this project is additive to her developments.
- I think one of two ways:
  1. Games in which the mechanics / controls build the requisite skills and muscle control needed for using a powered prosthesis.
  2. Games that educate and help new users become accustomed / familiar with their device / overcome stigmas around their device / embody their device
- Can be any, depends on the audience and what core mechanics can encourage use of the prosthetics.
- Since the attractiveness of a game is very subjective, I think that some principle game-mechanics needs to be found that are helpful for training functional prosthesis use. These mechanics should than be incorporated in a series of games that can be customised to individual preferences.
- Puzzle games
- I have no strong opinions
- One that trains the user to actually control their prosthesis, even if the task does not seem to be controlling a prosthesis
- 1. user-specific, it would be ideal if the users can set the environment the like to play in
- abstract, signals represented by menacing or rewarding objects and creatures. maybe. I am no expert here.

2.3.4 What hopes/expectations would you have for such a training game?

Limb Different:

- I think I would maybe try it.
- To be able to use my arm and hand to it's full potential, better muscles etc
- don't know
- I think a fighting game, that allows the use of a prosthetic that carried various weapons would be fun?
- Repetition becomes habit
- Not sure but perhaps if it could help with dexterity
- if there would be potential for reducing phantom limb pain that would be wonderful.
- Highlighting the potential of my ability to use advanced prosthetics-
- A reduction in wasted costs of unused prothetics for the NHS"
- To be able to use a prosthetic
- Actually no expectations as really don't know what can reasonably be expected!! Other than improved command of the robotic hand, and some enjoyment/fun in the process. I hope the games would be easy to use.
- Movement
- All
- Hand eye Coordination
- To get my brain and the muscles in my amputation site working it would really help me.
- To improve muscle tone & brain to nerve/muscle control

Researchers:

- That it is well grounded in the needs of patients and clinicians.
- Finding something that is appealing to a wide range of users; providing a setup that is easy to use and robust; ensuring a game framework that allows people to use their own devices; data protection is important if data will be shared.
- That it would accelerate learning of a new prosthesis; that users would enjoy using it; that it could provide wider benefit to other users or researchers (by data sharing).
- Easy to use, motivating, good transfer of skills to real prosthesis use
- That the game would be so immersive that the participant would not realise they are training their remnant muscles for optimal EMG based control.
- Long term engagement – not just a novelty that wears off after initial use
  Can be shared by prosthesis users and able-bodied players"
- the very least it must be engaging
- I think it is important for such games that they train the performing of activities of daily living with the prosthesis. Of course the games should be fun and attractive. However, the games should use individualised feedback to improve the functional use of the prosthesis. In the end the games should also relieve the therapists from time spending on rehabilitation. Moreover, the games should be helpful as an assessment tool for the appropriateness of a prosthesis for a given patient.
- That they would use it to build muscle and prevent fatigue.
- It should make data collection for training neuromuscular decoders fun so that we get more and better data to train our algorithms.
- To engage user and promote good technique
- a good game, 1. increases the the user engagement, 2. don't require clinical input frequently
- that the skills the user acquires then TRANSLATE to easier prosthetic usage

2.3.5 Challenges: In your opinion, what are the current challenges in terms of using games in prosthetics? & What are specific actions that could be taken by us as a community to address these challenges?

Challenges	Actions
Assessment and providing evidence that the game is ”working”.	We have to move towards more longitudinal studies.
Finding something that is appealing to a wide range of users; providing a setup that is easy to use and robust; ensuring a game framework that allows people to use their own devices; data protection is important if data will be shared.	Collating the wishes and experiences of a wide range of users; sharing knowledge, code and data; agreeing on a useful set of metrics to track learning.
transfer of skills to real prosthesis use	To combine the development of the game with research testing the transfer capacities. Ask patients to help with the development
Making the control imperceptible, yet related to the movements that are wanted for natural prosthetic control.	Large cohort study.
[No entry]	[No entry]
ability of the user limitation and level of engagement	Involve game developers when developing games
The biggest challenge is to have games that train functional prosthesis use. I think practically every game can be learned, also when it is controlled with myo-signals. However, most game studies focus only on in-game improvement. Now in-game improvement is a requirement for transfer to daily life performance. However, in-game improvement is not a sufficient requirement for transfer.<br>The challenge is that this point needs to be recognised in the broader community, and it deserves attention in each gaming paper.<br>I know this is me on my high horse, but I really think this is an important issue that is too often neglected. Most papers are written in a fashion that they assume that if in-game improvement is found, also transfer to prosthesis use occurs, but I doubt that.	Perform studies that actually show that only specific games show transfer from in-game performance to actual prosthesis use.
Applying electrodes.	Develop new electrodes.
Some categories of people and some age groups may associate ”games” with something frivolous and a waste of time.	Awareness. Educating participants about serious games and why the time they spend playing is well spent as it provides us with important data.
That playing a game teaches the users bad habits to win the games rather than to control their prosthesis	Understand the problem. How do we control our limbs/prostheses in the real world?
Lack of methods to create and evaluate evidence for the long term benefit of games when contrasted to other rehab methods.<br>Lack of games that actually could make any difference	Development of a platform to share knowledge and info about games in the prosthetics control
the field is very young, I don’t exactly know the answer here.	study psychology and design!
