# Supplementary figures and images for: Perception of Game-Based Rehabilitation in Upper Limb Prosthetic Training: Survey of Users and Researchers
Source: JMIR Serious Games. 2021 Feb 1;9(1):e23710. doi: 10.2196/23710 (PMC7884217; doi:10.2196/23710)

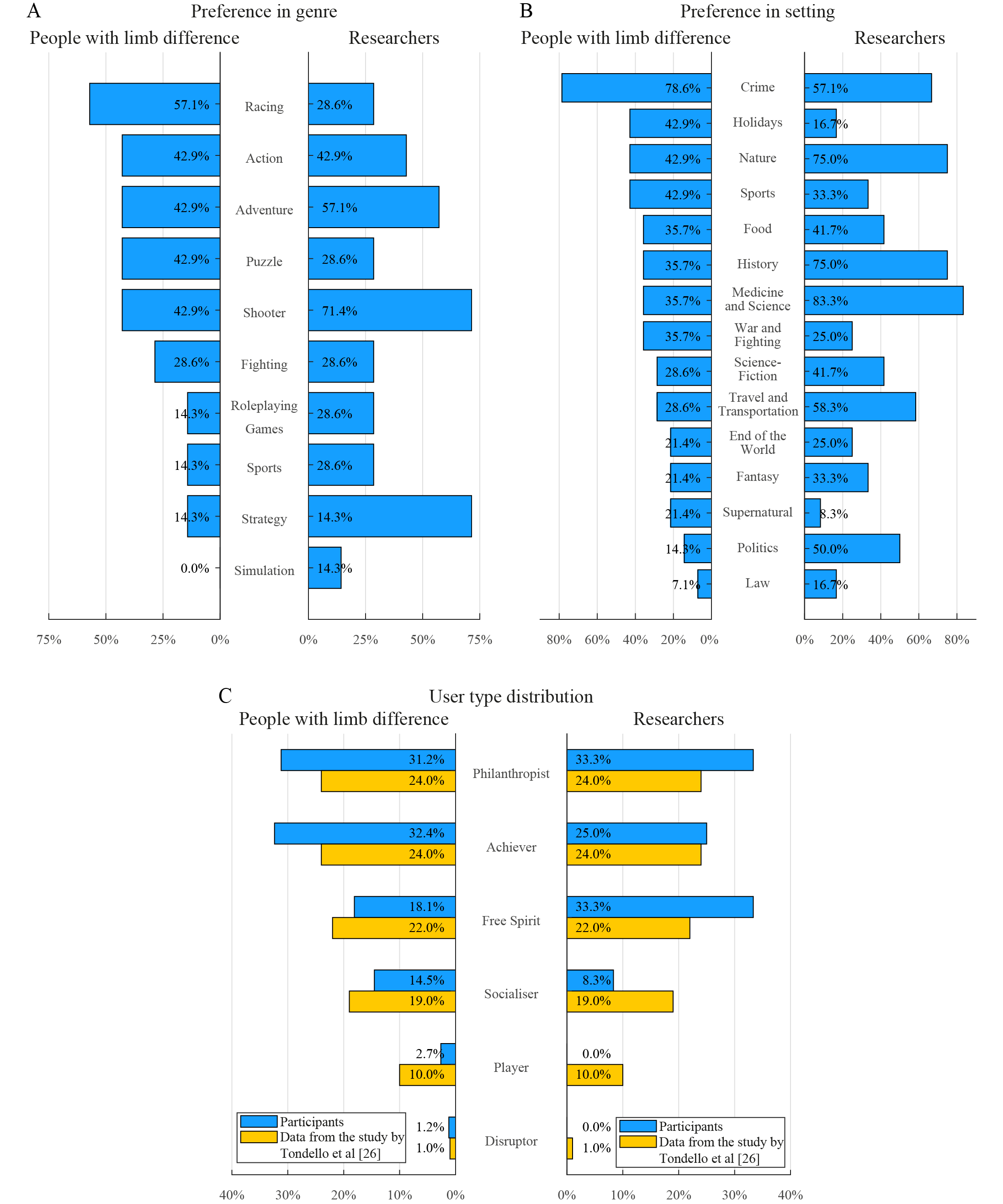

Supplement: Multimedia Appendix 2 [file games_v9i1e23710_app2.png]
